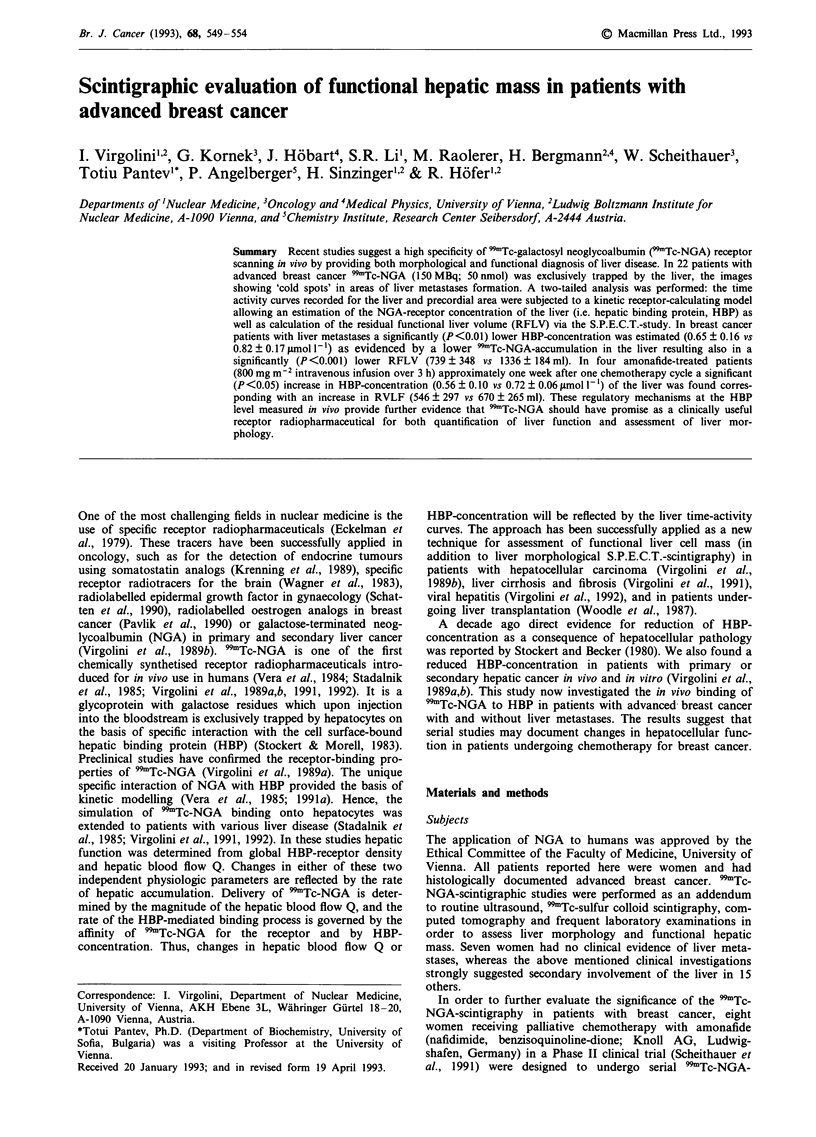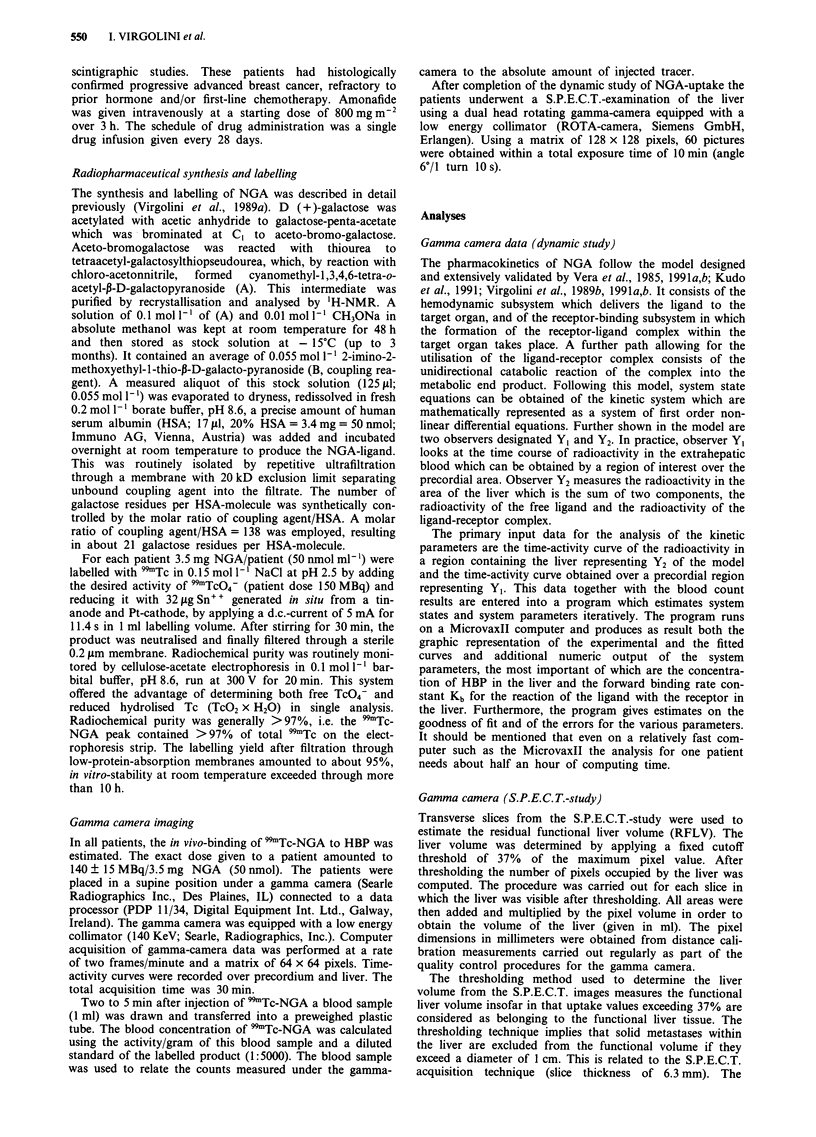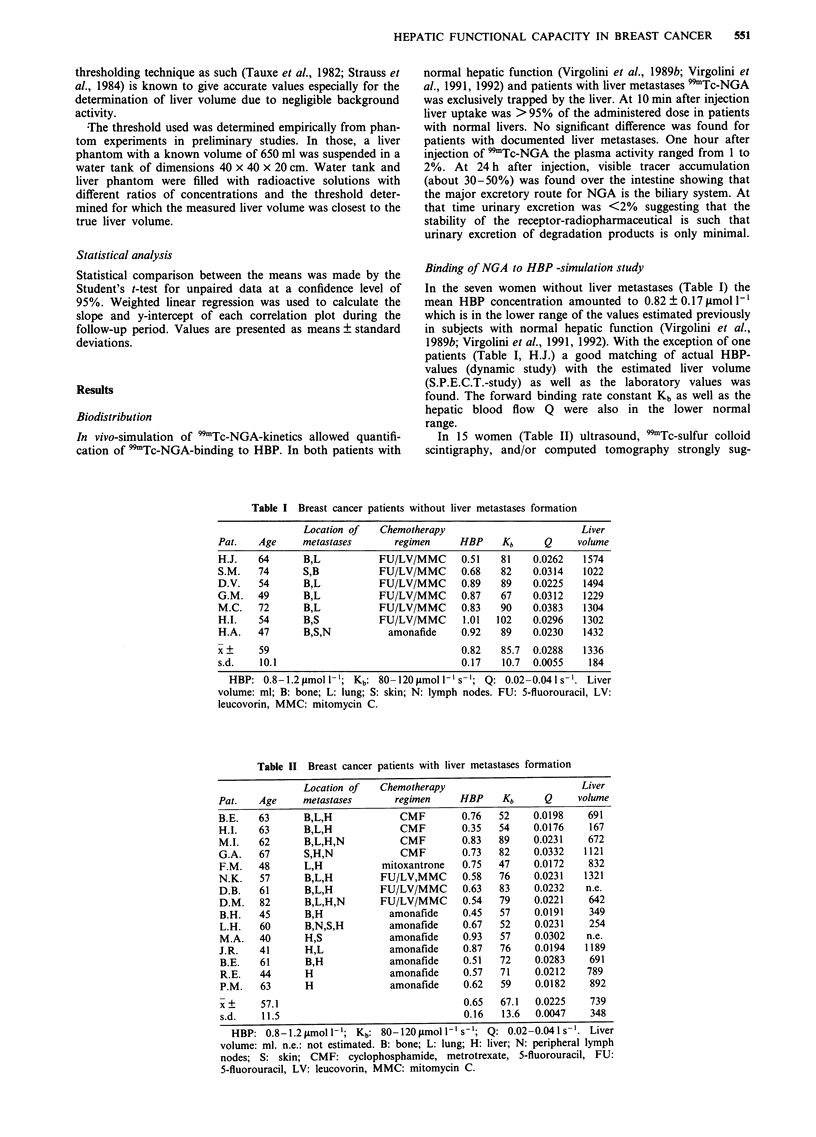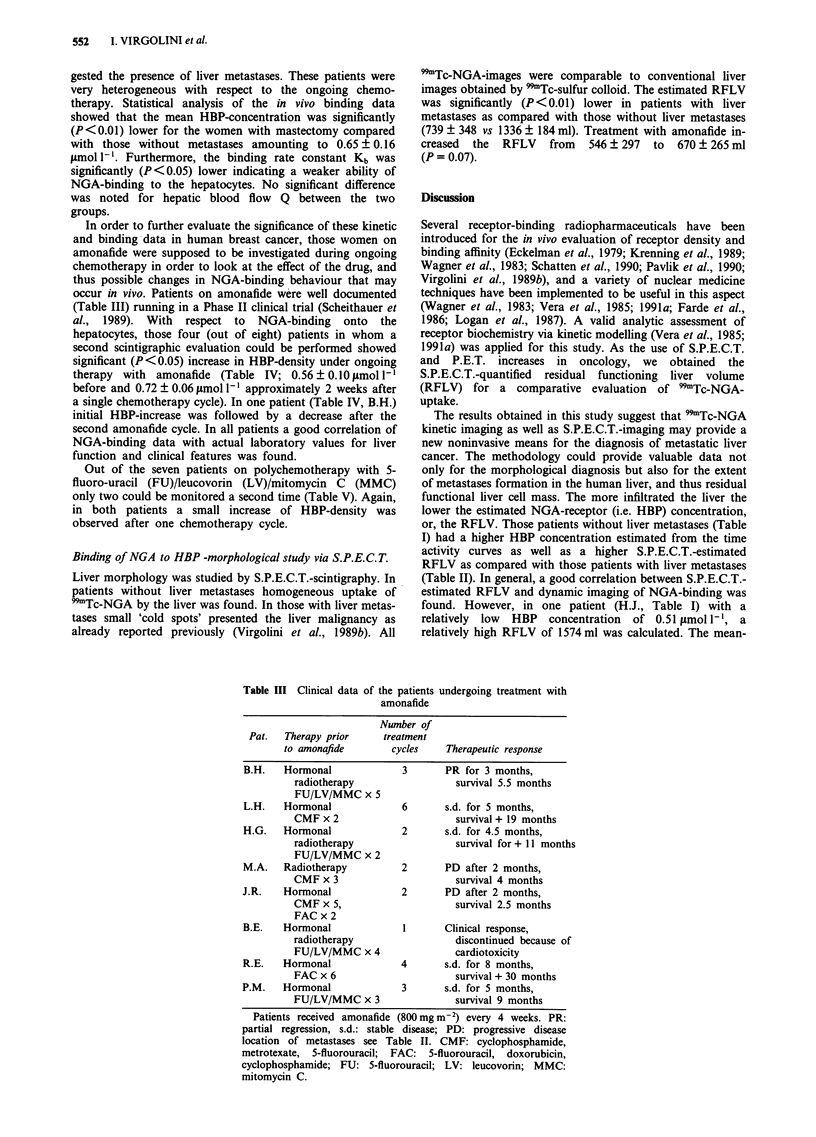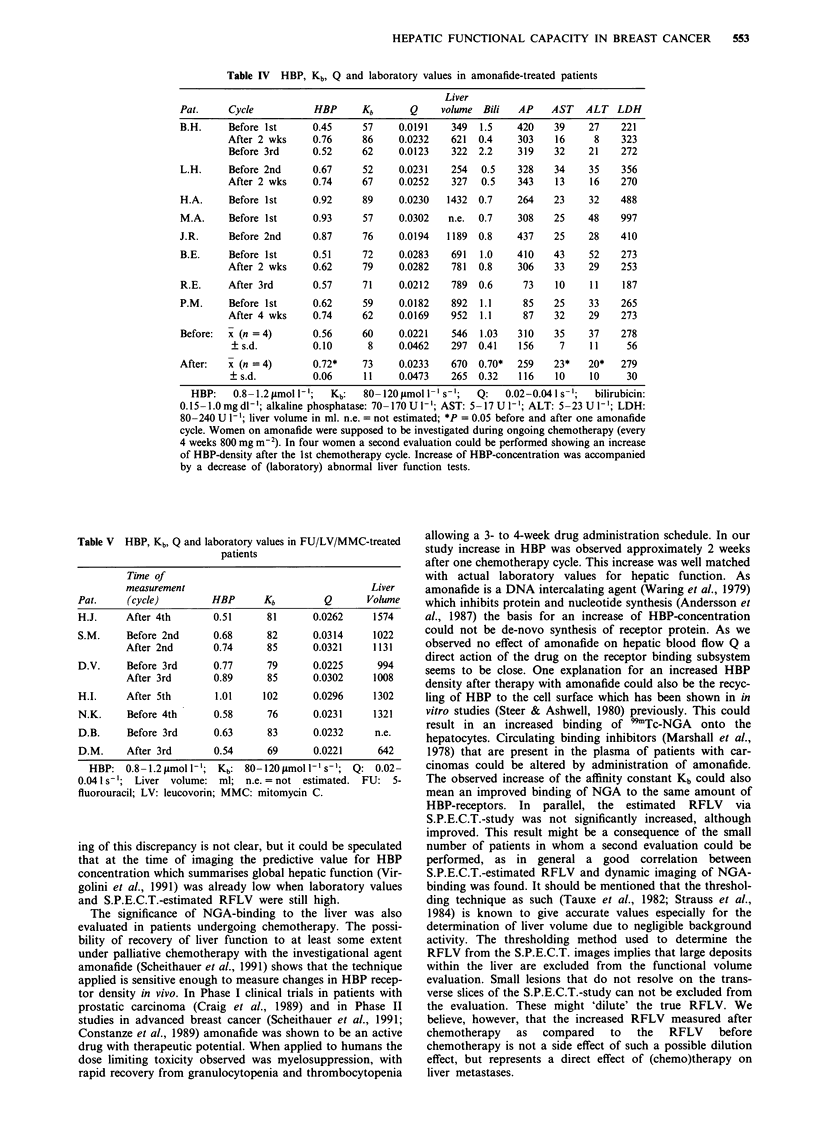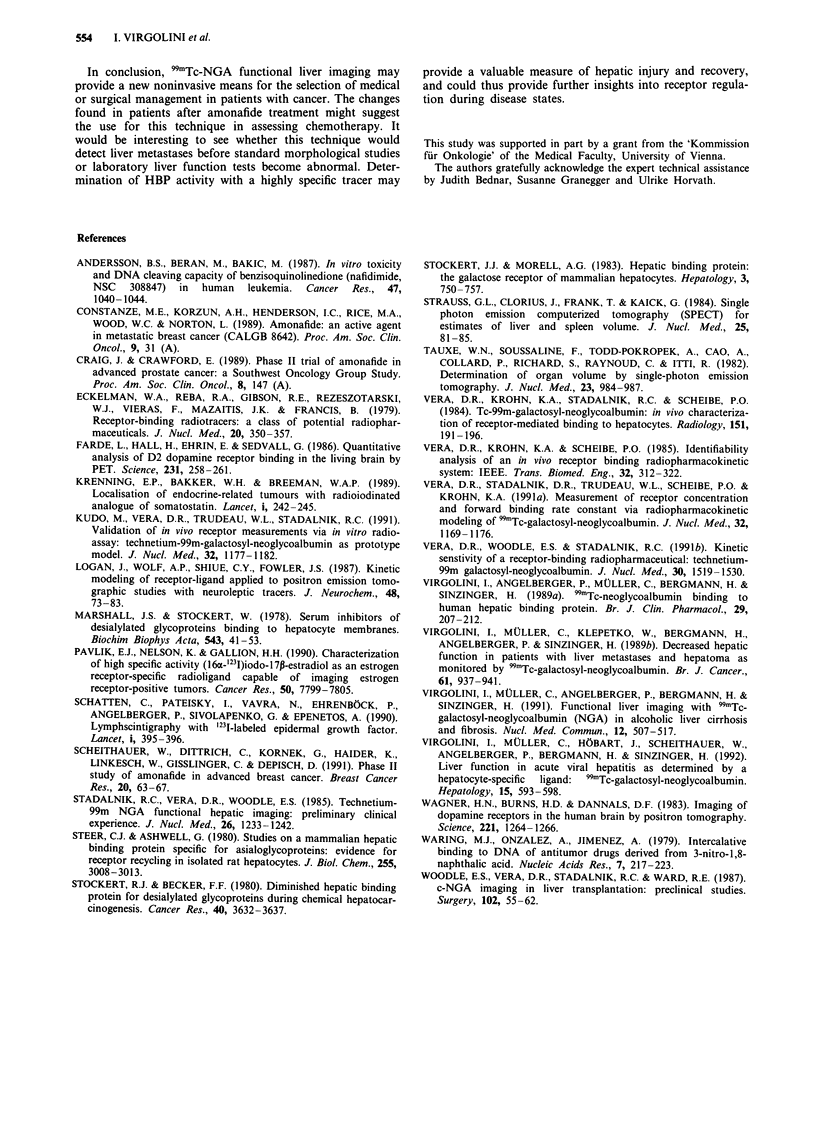# Scintigraphic evaluation of functional hepatic mass in patients with advanced breast cancer.

**DOI:** 10.1038/bjc.1993.384

**Published:** 1993-09

**Authors:** I. Virgolini, G. Kornek, J. Höbart, S. R. Li, M. Raolerer, H. Bergmann, W. Scheithauer, T. Pantev, P. Angelberger, H. Sinzinger

**Affiliations:** Department of Nuclear Medicine, University of Vienna, Austria.

## Abstract

Recent studies suggest a high specificity of 99mTc-galactosyl neoglycoalbumin (99mTc-NGA) receptor scanning in vivo by providing both morphological and functional diagnosis of liver disease. In 22 patients with advanced breast cancer 99mTc-NGA (150 MBq; 50 nmol) was exclusively trapped by the liver, the images showing 'cold spots' in areas of liver metastases formation. A two-tailed analysis was performed: the time activity curves recorded for the liver and precordial area were subjected to a kinetic receptor-calculating model allowing an estimation of the NGA-receptor concentration of the liver (i.e. hepatic binding protein, HBP) as well as calculation of the residual functional liver volume (RFLV) via the S.P.E.C.T.-study. In breast cancer patients with liver metastases a significantly (P < 0.01) lower HBP-concentration was estimated (0.65 +/- 0.16 vs 0.82 +/- 0.17 mumol l-1) as evidenced by a lower 99mTc-NGA-accumulation in the liver resulting also in a significantly (P < 0.001) lower RFLV (739 +/- 348 vs 1336 +/- 184 ml). In four amonafide-treated patients (800 mg m-2 intravenous infusion over 3 h) approximately one week after one chemotherapy cycle a significant (P < 0.05) increase in HBP-concentration (0.56 +/- 0.10 vs 0.72 +/- 0.06 mumol l-1) of the liver was found corresponding with an increase in RVLF (546 +/- 297 vs 670 +/- 265 ml). These regulatory mechanisms at the HBP level measured in vivo provide further evidence that 99mTc-NGA should have promise as a clinically useful receptor radiopharmaceutical for both quantification of liver function and assessment of liver morphology.


Br. J. Cancer (1993), 68, 549–554 © Macmillan Press Ltd., 1993

### Scintigraphic evaluation of functional hepatic mass in patients with advanced breast cancer

I. Virgolini[1,2], G. Kornek[3], J. Höbart[4], S.R. Li[1], M. Raolerer, H. Bergmann[2,4], W. Scheithauer[3], Totiu Pantev[1*], P. Angelberger[5], H. Sinzinger[1,2] & R. Höfer[1,2]

*Departments of [1]Nuclear Medicine, [3]Oncology and [4]Medical Physics, University of Vienna, [2]Ludwig Boltzmann Institute for Nuclear Medicine, A-1090 Vienna, and [5]Chemistry Institute, Research Center Seibersdorf, A-2444 Austria.*

**Summary** Recent studies suggest a high specificity of $^{99m}$Tc-galactosyl neoglycoalbumin ($^{99m}$Tc-NGA) receptor scanning *in vivo* by providing both morphological and functional diagnosis of liver disease. In 22 patients with advanced breast cancer $^{99m}$Tc-NGA (150 MBq; 50 nmol) was exclusively trapped by the liver, the images showing 'cold spots' in areas of liver metastases formation. A two-tailed analysis was performed: the time activity curves recorded for the liver and precordial area were subjected to a kinetic receptor-calculating model allowing an estimation of the NGA-receptor concentration of the liver (i.e. hepatic binding protein, HBP) as well as calculation of the residual functional liver volume (RFLV) via the S.P.E.C.T.-study. In breast cancer patients with liver metastases a significantly ($P$<0.01) lower HBP-concentration was estimated (0.65 ± 0.16 *vs* 0.82 ± 0.17 $\mu$mol l$^{-1}$) as evidenced by a lower $^{99m}$Tc-NGA-accumulation in the liver resulting also in a significantly ($P$<0.001) lower RFLV (739 ± 348 *vs* 1336 ± 184 ml). In four amonafide-treated patients (800 mg m$^{-2}$ intravenous infusion over 3 h) approximately one week after one chemotherapy cycle a significant ($P$<0.05) increase in HBP-concentration (0.56 ± 0.10 *vs* 0.72 ± 0.06 $\mu$mol l$^{-1}$) of the liver was found corresponding with an increase in RVLF (546 ± 297 *vs* 670 ± 265 ml). These regulatory mechanisms at the HBP level measured *in vivo* provide further evidence that $^{99m}$Tc-NGA should have promise as a clinically useful receptor radiopharmaceutical for both quantification of liver function and assessment of liver morphology.

One of the most challenging fields in nuclear medicine is the use of specific receptor radiopharmaceuticals (Eckelman *et al.*, 1979). These tracers have been successfully applied in oncology, such as for the detection of endocrine tumours using somatostatin analogs (Krenning *et al.*, 1989), specific receptor radiotracers for the brain (Wagner *et al.*, 1983), radiolabelled epidermal growth factor in gynaecology (Schatten *et al.*, 1990), radiolabelled oestrogen analogs in breast cancer (Pavlik *et al.*, 1990) or galactose-terminated neoglycoalbumin (NGA) in primary and secondary liver cancer (Virgolini *et al.*, 1989*b*). $^{99m}$Tc-NGA is one of the first chemically synthetised receptor radiopharmaceuticals introduced for *in vivo* use in humans (Vera *et al.*, 1984; Stadalnik *et al.*, 1985; Virgolini *et al.*, 1989*a*,*b*, 1991, 1992). It is a glycoprotein with galactose residues which upon injection into the bloodstream is exclusively trapped by hepatocytes on the basis of specific interaction with the cell surface-bound hepatic binding protein (HBP) (Stockert & Morell, 1983). Preclinical studies have confirmed the receptor-binding properties of $^{99m}$Tc-NGA (Virgolini *et al.*, 1989*a*). The unique specific interaction of NGA with HBP provided the basis of kinetic modelling (Vera *et al.*, 1985; 1991*a*). Hence, the simulation of $^{99m}$Tc-NGA binding onto hepatocytes was extended to patients with various liver disease (Stadalnik *et al.*, 1985; Virgolini *et al.*, 1991, 1992). In these studies hepatic function was determined from global HBP-receptor density and hepatic blood flow Q. Changes in either of these two independent physiologic parameters are reflected by the rate of hepatic accumulation. Delivery of $^{99m}$Tc-NGA is determined by the magnitude of the hepatic blood flow Q, and the rate of the HBP-mediated binding process is governed by the affinity of $^{99m}$Tc-NGA for the receptor and by HBP-concentration. Thus, changes in hepatic blood flow Q or HBP-concentration will be reflected by the liver time-activity curves. The approach has been successfully applied as a new technique for assessment of functional liver cell mass (in addition to liver morphological S.P.E.C.T.-scintigraphy) in patients with hepatocellular carcinoma (Virgolini *et al.*, 1989*b*), liver cirrhosis and fibrosis (Virgolini *et al.*, 1991), viral hepatitis (Virgolini *et al.*, 1992), and in patients undergoing liver transplantation (Woodle *et al.*, 1987).

A decade ago direct evidence for reduction of HBP-concentration as a consequence of hepatocellular pathology was reported by Stockert and Becker (1980). We also found a reduced HBP-concentration in patients with primary or secondary hepatic cancer *in vivo* and *in vitro* (Virgolini *et al.*, 1989*a*,*b*). This study now investigated the *in vivo* binding of $^{99m}$Tc-NGA to HBP in patients with advanced breast cancer with and without liver metastases. The results suggest that serial studies may document changes in hepatocellular function in patients undergoing chemotherapy for breast cancer.

### Materials and methods

### *Subjects*

The application of NGA to humans was approved by the Ethical Committee of the Faculty of Medicine, University of Vienna. All patients reported here were women and had histologically documented advanced breast cancer. $^{99m}$Tc-NGA-scintigraphic studies were performed as an addendum to routine ultrasound, $^{99m}$Tc-sulfur colloid scintigraphy, computed tomography and frequent laboratory examinations in order to assess liver morphology and functional hepatic mass. Seven women had no clinical evidence of liver metastases, whereas the above mentioned clinical investigations strongly suggested secondary involvement of the liver in 15 others.

In order to further evaluate the significance of the $^{99m}$Tc-NGA-scintigraphy in patients with breast cancer, eight women receiving palliative chemotherapy with amonafide (nafidimide, benzisoquinoline-dione; Knoll AG, Ludwigshafen, Germany) in a Phase II clinical trial (Scheithauer *et al.*, 1991) were designed to undergo serial $^{99m}$Tc-NGA-

Correspondence: I. Virgolini, Department of Nuclear Medicine, University of Vienna, AKH Ebene 3L, Währinger Gürtel 18–20, A-1090 Vienna, Austria.

*Totui Pantev, Ph.D. (Department of Biochemistry, University of Sofia, Bulgaria) was a visiting Professor at the University of Vienna.

Received 20 January 1993; and in revised form 19 April 1993.

scintigraphic studies. These patients had histologically confirmed progressive advanced breast cancer, refractory to prior hormone and/or first-line chemotherapy. Amonafide was given intravenously at a starting dose of 800 mg m$^{-2}$ over 3 h. The schedule of drug administration was a single drug infusion given every 28 days.

*Radiopharmaceutical synthesis and labelling*

The synthesis and labelling of NGA was described in detail previously (Virgolini *et al.*, 1989*a*). D (+)-galactose was acetylated with acetic anhydride to galactose-penta-acetate which was brominated at $C_1$ to aceto-bromo-galactose. Aceto-bromogalactose was reacted with thiourea to tetraacetyl-galactosylthiopseudourea, which, by reaction with chloro-acetonnitrile, formed cyanomethyl-1,3,4,6-tetra-*o*-acetyl-β-D-galactopyranoside (A). This intermediate was purified by recrystallisation and analysed by $^1$H-NMR. A solution of 0.1 mol l$^{-1}$ of (A) and 0.01 mol l$^{-1}$ $CH_3ONa$ in absolute methanol was kept at room temperature for 48 h and then stored as stock solution at − 15°C (up to 3 months). It contained an average of 0.055 mol l$^{-1}$ 2-imino-2-methoxyethyl-1-thio-β-D-galacto-pyranoside (B, coupling reagent). A measured aliquot of this stock solution (125 µl; 0.055 mol l$^{-1}$) was evaporated to dryness, redissolved in fresh 0.2 mol l$^{-1}$ borate buffer, pH 8.6, a precise amount of human serum albumin (HSA; 17 µl, 20% HSA = 3.4 mg = 50 nmol; Immuno AG, Vienna, Austria) was added and incubated overnight at room temperature to produce the NGA-ligand. This was routinely isolated by repetitive ultrafiltration through a membrane with 20 kD exclusion limit separating unbound coupling agent into the filtrate. The number of galactose residues per HSA-molecule was synthetically controlled by the molar ratio of coupling agent/HSA. A molar ratio of coupling agent/HSA = 138 was employed, resulting in about 21 galactose residues per HSA-molecule.

For each patient 3.5 mg NGA/patient (50 nmol ml$^{-1}$) were labelled with $^{99m}$Tc in 0.15 mol l$^{-1}$ NaCl at pH 2.5 by adding the desired activity of $^{99m}TcO_4^-$ (patient dose 150 MBq) and reducing it with 32 µg $Sn^{++}$ generated *in situ* from a tin-anode and Pt-cathode, by applying a d.c.-current of 5 mA for 11.4 s in 1 ml labelling volume. After stirring for 30 min, the product was neutralised and finally filtered through a sterile 0.2 µm membrane. Radiochemical purity was routinely monitored by cellulose-acetate electrophoresis in 0.1 mol l$^{-1}$ barbital buffer, pH 8.6, run at 300 V for 20 min. This system offered the advantage of determining both free $TcO_4^-$ and reduced hydrolised Tc ($TcO_2 \times H_2O$) in single analysis. Radiochemical purity was generally >97%, i.e. the $^{99m}$Tc-NGA peak contained >97% of total $^{99m}$Tc on the electrophoresis strip. The labelling yield after filtration through low-protein-absorption membranes amounted to about 95%, *in vitro*-stability at room temperature exceeded through more than 10 h.

*Gamma camera imaging*

In all patients, the *in vivo*-binding of $^{99m}$Tc-NGA to HBP was estimated. The exact dose given to a patient amounted to 140 ± 15 MBq/3.5 mg NGA (50 nmol). The patients were placed in a supine position under a gamma camera (Searle Radiographics Inc., Des Plaines, IL) connected to a data processor (PDP 11/34, Digital Equipment Int. Ltd., Galway, Ireland). The gamma camera was equipped with a low energy collimator (140 KeV; Searle, Radiographics, Inc.). Computer acquisition of gamma-camera data was performed at a rate of two frames/minute and a matrix of 64 × 64 pixels. Time-activity curves were recorded over precordium and liver. The total acquisition time was 30 min.

Two to 5 min after injection of $^{99m}$Tc-NGA a blood sample (1 ml) was drawn and transferred into a preweighed plastic tube. The blood concentration of $^{99m}$Tc-NGA was calculated using the activity/gram of this blood sample and a diluted standard of the labelled product (1:5000). The blood sample was used to relate the counts measured under the gamma-camera to the absolute amount of injected tracer.

After completion of the dynamic study of NGA-uptake the patients underwent a S.P.E.C.T.-examination of the liver using a dual head rotating gamma-camera equipped with a low energy collimator (ROTA-camera, Siemens GmbH, Erlangen). Using a matrix of 128 × 128 pixels, 60 pictures were obtained within a total exposure time of 10 min (angle 6°/1 turn 10 s).

### Analyses

*Gamma camera data (dynamic study)*

The pharmacokinetics of NGA follow the model designed and extensively validated by Vera *et al.*, 1985, 1991*a*,*b*; Kudo *et al.*, 1991; Virgolini *et al.*, 1989*b*, 1991*a*,*b*. It consists of the hemodynamic subsystem which delivers the ligand to the target organ, and of the receptor-binding subsystem in which the formation of the receptor-ligand complex within the target organ takes place. A further path allowing for the utilisation of the ligand-receptor complex consists of the unidirectional catabolic reaction of the complex into the metabolic end product. Following this model, system state equations can be obtained of the kinetic system which are mathematically represented as a system of first order non-linear differential equations. Further shown in the model are two observers designated $Y_1$ and $Y_2$. In practice, observer $Y_1$ looks at the time course of radioactivity in the extrahepatic blood which can be obtained by a region of interest over the precordial area. Observer $Y_2$ measures the radioactivity in the area of the liver which is the sum of two components, the radioactivity of the free ligand and the radioactivity of the ligand-receptor complex.

The primary input data for the analysis of the kinetic parameters are the time-activity curve of the radioactivity in a region containing the liver representing $Y_2$ of the model and the time-activity curve obtained over a precordial region representing $Y_1$. This data together with the blood count results are entered into a program which estimates system states and system parameters iteratively. The program runs on a MicrovaxII computer and produces as result both the graphic representation of the experimental and the fitted curves and additional numeric output of the system parameters, the most important of which are the concentration of HBP in the liver and the forward binding rate constant $K_b$ for the reaction of the ligand with the receptor in the liver. Furthermore, the program gives estimates on the goodness of fit and of the errors for the various parameters. It should be mentioned that even on a relatively fast computer such as the MicrovaxII the analysis for one patient needs about half an hour of computing time.

*Gamma camera (S.P.E.C.T.-study)*

Transverse slices from the S.P.E.C.T.-study were used to estimate the residual functional liver volume (RFLV). The liver volume was determined by applying a fixed cutoff threshold of 37% of the maximum pixel value. After thresholding the number of pixels occupied by the liver was computed. The procedure was carried out for each slice in which the liver was visible after thresholding. All areas were then added and multiplied by the pixel volume in order to obtain the volume of the liver (given in ml). The pixel dimensions in millimeters were obtained from distance calibration measurements carried out regularly as part of the quality control procedures for the gamma camera.

The thresholding method used to determine the liver volume from the S.P.E.C.T. images measures the functional liver volume insofar in that uptake values exceeding 37% are considered as belonging to the functional liver tissue. The thresholding technique implies that solid metastases within the liver are excluded from the functional volume if they exceed a diameter of 1 cm. This is related to the S.P.E.C.T. acquisition technique (slice thickness of 6.3 mm). The thresholding technique as such (Tauxe *et al.*, 1982; Strauss *et al.*, 1984) is known to give accurate values especially for the determination of liver volume due to negligible background activity.

The threshold used was determined empirically from phantom experiments in preliminary studies. In those, a liver phantom with a known volume of 650 ml was suspended in a water tank of dimensions 40 × 40 × 20 cm. Water tank and liver phantom were filled with radioactive solutions with different ratios of concentrations and the threshold determined for which the measured liver volume was closest to the true liver volume.

*Statistical analysis*

Statistical comparison between the means was made by the Student's *t*-test for unpaired data at a confidence level of 95%. Weighted linear regression was used to calculate the slope and y-intercept of each correlation plot during the follow-up period. Values are presented as means ± standard deviations.

### Results

*Biodistribution*

*In vivo*-simulation of $^{99m}$Tc-NGA-kinetics allowed quantification of $^{99m}$Tc-NGA-binding to HBP. In both patients with normal hepatic function (Virgolini *et al.*, 1989*b*; Virgolini *et al.*, 1991, 1992) and patients with liver metastases $^{99m}$Tc-NGA was exclusively trapped by the liver. At 10 min after injection liver uptake was >95% of the administered dose in patients with normal livers. No significant difference was found for patients with documented liver metastases. One hour after injection of $^{99m}$Tc-NGA the plasma activity ranged from 1 to 2%. At 24 h after injection, visible tracer accumulation (about 30–50%) was found over the intestine showing that the major excretory route for NGA is the biliary system. At that time urinary excretion was <2% suggesting that the stability of the receptor-radiopharmaceutical is such that urinary excretion of degradation products is only minimal.

*Binding of NGA to HBP -simulation study*

In the seven women without liver metastases (Table I) the mean HBP concentration amounted to 0.82 ± 0.17 μmol $l^{-1}$ which is in the lower range of the values estimated previously in subjects with normal hepatic function (Virgolini *et al.*, 1989*b*; Virgolini *et al.*, 1991, 1992). With the exception of one patients (Table I, H.J.) a good matching of actual HBP-values (dynamic study) with the estimated liver volume (S.P.E.C.T.-study) as well as the laboratory values was found. The forward binding rate constant $K_b$ as well as the hepatic blood flow Q were also in the lower normal range.

In 15 women (Table II) ultrasound, $^{99m}$Tc-sulfur colloid scintigraphy, and/or computed tomography strongly sug-

**Table I** Breast cancer patients without liver metastases formation

| *Pat.* | *Age* | *Location of metastases* | *Chemotherapy regimen* | *HBP* | $K_b$ | *Q* | *Liver volume* |
|---|---|---|---|---|---|---|---|
| H.J. | 64 | B,L | FU/LV/MMC | 0.51 | 81 | 0.0262 | 1574 |
| S.M. | 74 | S,B | FU/LV/MMC | 0.68 | 82 | 0.0314 | 1022 |
| D.V. | 54 | B,L | FU/LV/MMC | 0.89 | 89 | 0.0225 | 1494 |
| G.M. | 49 | B,L | FU/LV/MMC | 0.87 | 67 | 0.0312 | 1229 |
| M.C. | 72 | B,L | FU/LV/MMC | 0.83 | 90 | 0.0383 | 1304 |
| H.I. | 54 | B,S | FU/LV/MMC | 1.01 | 102 | 0.0296 | 1302 |
| H.A. | 47 | B,S,N | amonafide | 0.92 | 89 | 0.0230 | 1432 |
| $\bar{x}$ ± | 59 | | | 0.82 | 85.7 | 0.0288 | 1336 |
| s.d. | 10.1 | | | 0.17 | 10.7 | 0.0055 | 184 |

HBP: 0.8–1.2 μmol $l^{-1}$; $K_b$: 80–120 μmol $l^{-1}$ $s^{-1}$; Q: 0.02–0.04 l $s^{-1}$. Liver volume: ml; B: bone; L: lung; S: skin; N: lymph nodes. FU: 5-fluorouracil, LV: leucovorin, MMC: mitomycin C.

**Table II** Breast cancer patients with liver metastases formation

| *Pat.* | *Age* | *Location of metastases* | *Chemotherapy regimen* | *HBP* | $K_b$ | *Q* | *Liver volume* |
|---|---|---|---|---|---|---|---|
| B.E. | 63 | B,L,H | CMF | 0.76 | 52 | 0.0198 | 691 |
| H.I. | 63 | B,L,H | CMF | 0.35 | 54 | 0.0176 | 167 |
| M.I. | 62 | B,L,H,N | CMF | 0.83 | 89 | 0.0231 | 672 |
| G.A. | 67 | S,H,N | CMF | 0.73 | 82 | 0.0332 | 1121 |
| F.M. | 48 | L,H | mitoxantrone | 0.75 | 47 | 0.0172 | 832 |
| N.K. | 57 | B,L,H | FU/LV,MMC | 0.58 | 76 | 0.0231 | 1321 |
| D.B. | 61 | B,L,H | FU/LV/MMC | 0.63 | 83 | 0.0232 | n.e. |
| D.M. | 82 | B,L,H,N | FU/LV/MMC | 0.54 | 79 | 0.0221 | 642 |
| B.H. | 45 | B,H | amonafide | 0.45 | 57 | 0.0191 | 349 |
| L.H. | 60 | B,N,S,H | amonafide | 0.67 | 52 | 0.0231 | 254 |
| M.A. | 40 | H,S | amonafide | 0.93 | 57 | 0.0302 | n.e. |
| J.R. | 41 | H,L | amonafide | 0.87 | 76 | 0.0194 | 1189 |
| B.E. | 61 | B,H | amonafide | 0.51 | 72 | 0.0283 | 691 |
| R.E. | 44 | H | amonafide | 0.57 | 71 | 0.0212 | 789 |
| P.M. | 63 | H | amonafide | 0.62 | 59 | 0.0182 | 892 |
| $\bar{x}$ ± | 57.1 | | | 0.65 | 67.1 | 0.0225 | 739 |
| s.d. | 11.5 | | | 0.16 | 13.6 | 0.0047 | 348 |

HBP: 0.8–1.2 μmol $l^{-1}$; $K_b$: 80–120 μmol $l^{-1}$ $s^{-1}$; Q: 0.02–0.04 l $s^{-1}$. Liver volume: ml. n.e.: not estimated. B: bone; L: lung; H: liver; N: peripheral lymph nodes; S: skin; CMF: cyclophosphamide, metrotrexate, 5-fluorouracil, FU: 5-fluorouracil, LV: leucovorin, MMC: mitomycin C.

gested the presence of liver metastases. These patients were very heterogeneous with respect to the ongoing chemotherapy. Statistical analysis of the *in vivo* binding data showed that the mean HBP-concentration was significantly ($P<0.01$) lower for the women with mastectomy compared with those without metastases amounting to $0.65 \pm 0.16$ $\mu mol\ l^{-1}$. Furthermore, the binding rate constant $K_b$ was significantly ($P<0.05$) lower indicating a weaker ability of NGA-binding to the hepatocytes. No significant difference was noted for hepatic blood flow Q between the two groups.

In order to further evaluate the significance of these kinetic and binding data in human breast cancer, those women on amonafide were supposed to be investigated during ongoing chemotherapy in order to look at the effect of the drug, and thus possible changes in NGA-binding behaviour that may occur *in vivo*. Patients on amonafide were well documented (Table III) running in a Phase II clinical trial (Scheithauer *et al.*, 1989). With respect to NGA-binding onto the hepatocytes, those four (out of eight) patients in whom a second scintigraphic evaluation could be performed showed significant ($P<0.05$) increase in HBP-density under ongoing therapy with amonafide (Table IV; $0.56 \pm 0.10\ \mu mol\ l^{-1}$ before and $0.72 \pm 0.06\ \mu mol\ l^{-1}$ approximately 2 weeks after a single chemotherapy cycle). In one patient (Table IV, B.H.) initial HBP-increase was followed by a decrease after the second amonafide cycle. In all patients a good correlation of NGA-binding data with actual laboratory values for liver function and clinical features was found.

Out of the seven patients on polychemotherapy with 5-fluoro-uracil (FU)/leucovorin (LV)/mitomycin C (MMC) only two could be monitored a second time (Table V). Again, in both patients a small increase of HBP-density was observed after one chemotherapy cycle.

*Binding of NGA to HBP -morphological study via S.P.E.C.T.*

Liver morphology was studied by S.P.E.C.T.-scintigraphy. In patients without liver metastases homogeneous uptake of $^{99m}$Tc-NGA by the liver was found. In those with liver metastases small 'cold spots' presented the liver malignancy as already reported previously (Virgolini *et al.*, 1989*b*). All $^{99m}$Tc-NGA-images were comparable to conventional liver images obtained by $^{99m}$Tc-sulfur colloid. The estimated RFLV was significantly ($P<0.01$) lower in patients with liver metastases as compared with those without liver metastases ($739 \pm 348$ *vs* $1336 \pm 184$ ml). Treatment with amonafide increased the RFLV from $546 \pm 297$ to $670 \pm 265$ ml ($P = 0.07$).

### Discussion

Several receptor-binding radiopharmaceuticals have been introduced for the *in vivo* evaluation of receptor density and binding affinity (Eckelman *et al.*, 1979; Krenning *et al.*, 1989; Wagner *et al.*, 1983; Schatten *et al.*, 1990; Pavlik *et al.*, 1990; Virgolini *et al.*, 1989*b*), and a variety of nuclear medicine techniques have been implemented to be useful in this aspect (Wagner *et al.*, 1983; Vera *et al.*, 1985; 1991*a*; Farde *et al.*, 1986; Logan *et al.*, 1987). A valid analytic assessment of receptor biochemistry via kinetic modelling (Vera *et al.*, 1985; 1991*a*) was applied for this study. As the use of S.P.E.C.T. and P.E.T. increases in oncology, we obtained the S.P.E.C.T.-quantified residual functioning liver volume (RFLV) for a comparative evaluation of $^{99m}$Tc-NGA-uptake.

The results obtained in this study suggest that $^{99m}$Tc-NGA kinetic imaging as well as S.P.E.C.T.-imaging may provide a new noninvasive means for the diagnosis of metastatic liver cancer. The methodology could provide valuable data not only for the morphological diagnosis but also for the extent of metastases formation in the human liver, and thus residual functional liver cell mass. The more infiltrated the liver the lower the estimated NGA-receptor (i.e. HBP) concentration, or, the RFLV. Those patients without liver metastases (Table I) had a higher HBP concentration estimated from the time activity curves as well as a higher S.P.E.C.T.-estimated RFLV as compared with those patients with liver metastases (Table II). In general, a good correlation between S.P.E.C.T.-estimated RFLV and dynamic imaging of NGA-binding was found. However, in one patient (H.J., Table I) with a relatively low HBP concentration of $0.51\ \mu mol\ l^{-1}$, a relatively high RFLV of 1574 ml was calculated. The mean-

**Table III** Clinical data of the patients undergoing treatment with amonafide

| *Pat.* | *Therapy prior to amonafide* | *Number of treatment cycles* | *Therapeutic response* |
|---|---|---|---|
| B.H. | Hormonal<br>radiotherapy<br>FU/LV/MMC × 5 | 3 | PR for 3 months,<br>survival 5.5 months |
| L.H. | Hormonal<br>CMF × 2 | 6 | s.d. for 5 months,<br>survival + 19 months |
| H.G. | Hormonal<br>radiotherapy<br>FU/LV/MMC × 2 | 2 | s.d. for 4.5 months,<br>survival for + 11 months |
| M.A. | Radiotherapy<br>CMF × 3 | 2 | PD after 2 months,<br>survival 4 months |
| J.R. | Hormonal<br>CMF × 5,<br>FAC × 2 | 2 | PD after 2 months,<br>survival 2.5 months |
| B.E. | Hormonal<br>radiotherapy<br>FU/LV/MMC × 4 | 1 | Clinical response,<br>discontinued because of<br>cardiotoxicity |
| R.E. | Hormonal<br>FAC × 6 | 4 | s.d. for 8 months,<br>survival + 30 months |
| P.M. | Hormonal<br>FU/LV/MMC × 3 | 3 | s.d. for 5 months,<br>survival 9 months |

Patients received amonafide (800 mg $m^{-2}$) every 4 weeks. PR: partial regression, s.d.: stable disease; PD: progressive disease location of metastases see Table II. CMF: cyclophosphamide, metrotexate, 5-fluorouracil; FAC: 5-fluorouracil, doxorubicin, cyclophosphamide; FU: 5-fluorouracil; LV: leucovorin; MMC: mitomycin C.

**Table IV** HBP, $K_b$, Q and laboratory values in amonafide-treated patients

| *Pat.* | *Cycle* | *HBP* | $K_b$ | *Q* | *Liver volume* | *Bili* | *AP* | *AST* | *ALT* | *LDH* |
|---|---|---|---|---|---|---|---|---|---|---|
| B.H. | Before 1st | 0.45 | 57 | 0.0191 | 349 | 1.5 | 420 | 39 | 27 | 221 |
| | After 2 wks | 0.76 | 86 | 0.0232 | 621 | 0.4 | 303 | 16 | 8 | 323 |
| | Before 3rd | 0.52 | 62 | 0.0123 | 322 | 2.2 | 319 | 32 | 21 | 272 |
| L.H. | Before 2nd | 0.67 | 52 | 0.0231 | 254 | 0.5 | 328 | 34 | 35 | 356 |
| | After 2 wks | 0.74 | 67 | 0.0252 | 327 | 0.5 | 343 | 13 | 16 | 270 |
| H.A. | Before 1st | 0.92 | 89 | 0.0230 | 1432 | 0.7 | 264 | 23 | 32 | 488 |
| M.A. | Before 1st | 0.93 | 57 | 0.0302 | n.e. | 0.7 | 308 | 25 | 48 | 997 |
| J.R. | Before 2nd | 0.87 | 76 | 0.0194 | 1189 | 0.8 | 437 | 25 | 28 | 410 |
| B.E. | Before 1st | 0.51 | 72 | 0.0283 | 691 | 1.0 | 410 | 43 | 52 | 273 |
| | After 2 wks | 0.62 | 79 | 0.0282 | 781 | 0.8 | 306 | 33 | 29 | 253 |
| R.E. | After 3rd | 0.57 | 71 | 0.0212 | 789 | 0.6 | 73 | 10 | 11 | 187 |
| P.M. | Before 1st | 0.62 | 59 | 0.0182 | 892 | 1.1 | 85 | 25 | 33 | 265 |
| | After 4 wks | 0.74 | 62 | 0.0169 | 952 | 1.1 | 87 | 32 | 29 | 273 |
| Before: | $\bar{x}$ ($n$ = 4) | 0.56 | 60 | 0.0221 | 546 | 1.03 | 310 | 35 | 37 | 278 |
| | ± s.d. | 0.10 | 8 | 0.0462 | 297 | 0.41 | 156 | 7 | 11 | 56 |
| After: | $\bar{x}$ ($n$ = 4) | 0.72* | 73 | 0.0233 | 670 | 0.70* | 259 | 23* | 20* | 279 |
| | ± s.d. | 0.06 | 11 | 0.0473 | 265 | 0.32 | 116 | 10 | 10 | 30 |

HBP: 0.8–1.2 $\mu$mol $l^{-1}$; $K_b$: 80–120 $\mu$mol $l^{-1}$ $s^{-1}$; Q: 0.02–0.04 l $s^{-1}$; bilirubicin: 0.15–1.0 mg $dl^{-1}$; alkaline phosphatase: 70–170 U $l^{-1}$; AST: 5–17 U $l^{-1}$; ALT: 5–23 U $l^{-1}$; LDH: 80–240 U $l^{-1}$; liver volume in ml. n.e. = not estimated; *$P$ = 0.05 before and after one amonafide cycle. Women on amonafide were supposed to be investigated during ongoing chemotherapy (every 4 weeks 800 mg $m^{-2}$). In four women a second evaluation could be performed showing an increase of HBP-density after the 1st chemotherapy cycle. Increase of HBP-concentration was accompanied by a decrease of (laboratory) abnormal liver function tests.

**Table V** HBP, $K_b$, Q and laboratory values in FU/LV/MMC-treated patients

| *Pat.* | *Time of measurement (cycle)* | *HBP* | $K_b$ | *Q* | *Liver Volume* |
|---|---|---|---|---|---|
| H.J. | After 4th | 0.51 | 81 | 0.0262 | 1574 |
| S.M. | Before 2nd | 0.68 | 82 | 0.0314 | 1022 |
| | After 2nd | 0.74 | 85 | 0.0321 | 1131 |
| D.V. | Before 3rd | 0.77 | 79 | 0.0225 | 994 |
| | After 3rd | 0.89 | 85 | 0.0302 | 1008 |
| H.I. | After 5th | 1.01 | 102 | 0.0296 | 1302 |
| N.K. | Before 4th | 0.58 | 76 | 0.0231 | 1321 |
| D.B. | Before 3rd | 0.63 | 83 | 0.0232 | n.e. |
| D.M. | After 3rd | 0.54 | 69 | 0.0221 | 642 |

HBP: 0.8–1.2 $\mu$mol $l^{-1}$; $K_b$: 80–120 $\mu$mol $l^{-1}$ $s^{-1}$; Q: 0.02–0.04 l $s^{-1}$; Liver volume: ml; n.e. = not estimated. FU: 5-fluorouracil; LV: leucovorin; MMC: mitomycin C.

ing of this discrepancy is not clear, but it could be speculated that at the time of imaging the predictive value for HBP concentration which summarises global hepatic function (Virgolini *et al.*, 1991) was already low when laboratory values and S.P.E.C.T.-estimated RFLV were still high.

The significance of NGA-binding to the liver was also evaluated in patients undergoing chemotherapy. The possibility of recovery of liver function to at least some extent under palliative chemotherapy with the investigational agent amonafide (Scheithauer *et al.*, 1991) shows that the technique applied is sensitive enough to measure changes in HBP receptor density *in vivo*. In Phase I clinical trials in patients with prostatic carcinoma (Craig *et al.*, 1989) and in Phase II studies in advanced breast cancer (Scheithauer *et al.*, 1991; Constanze *et al.*, 1989) amonafide was shown to be an active drug with therapeutic potential. When applied to humans the dose limiting toxicity observed was myelosuppression, with rapid recovery from granulocytopenia and thrombocytopenia allowing a 3- to 4-week drug administration schedule. In our study increase in HBP was observed approximately 2 weeks after one chemotherapy cycle. This increase was well matched with actual laboratory values for hepatic function. As amonafide is a DNA intercalating agent (Waring *et al.*, 1979) which inhibits protein and nucleotide synthesis (Andersson *et al.*, 1987) the basis for an increase of HBP-concentration could not be de-novo synthesis of receptor protein. As we observed no effect of amonafide on hepatic blood flow Q a direct action of the drug on the receptor binding subsystem seems to be close. One explanation for an increased HBP density after therapy with amonafide could also be the recycling of HBP to the cell surface which has been shown in *in vitro* studies (Steer & Ashwell, 1980) previously. This could result in an increased binding of $^{99m}$Tc-NGA onto the hepatocytes. Circulating binding inhibitors (Marshall *et al.*, 1978) that are present in the plasma of patients with carcinomas could be altered by administration of amonafide. The observed increase of the affinity constant $K_b$ could also mean an improved binding of NGA to the same amount of HBP-receptors. In parallel, the estimated RFLV via S.P.E.C.T.-study was not significantly increased, although improved. This result might be a consequence of the small number of patients in whom a second evaluation could be performed, as in general a good correlation between S.P.E.C.T.-estimated RFLV and dynamic imaging of NGA-binding was found. It should be mentioned that the thresholding technique as such (Tauxe *et al.*, 1982; Strauss *et al.*, 1984) is known to give accurate values especially for the determination of liver volume due to negligible background activity. The thresholding method used to determine the RFLV from the S.P.E.C.T. images implies that large deposits within the liver are excluded from the functional volume evaluation. Small lesions that do not resolve on the transverse slices of the S.P.E.C.T.-study can not be excluded from the evaluation. These might 'dilute' the true RFLV. We believe, however, that the increased RFLV measured after chemotherapy as compared to the RFLV before chemotherapy is not a side effect of such a possible dilution effect, but represents a direct effect of (chemo)therapy on liver metastases.

In conclusion, $^{99m}$Tc-NGA functional liver imaging may provide a new noninvasive means for the selection of medical or surgical management in patients with cancer. The changes found in patients after amonafide treatment might suggest the use for this technique in assessing chemotherapy. It would be interesting to see whether this technique would detect liver metastases before standard morphological studies or laboratory liver function tests become abnormal. Determination of HBP activity with a highly specific tracer may provide a valuable measure of hepatic injury and recovery, and could thus provide further insights into receptor regulation during disease states.

This study was supported in part by a grant from the 'Kommission für Onkologie' of the Medical Faculty, University of Vienna.

The authors gratefully acknowledge the expert technical assistance by Judith Bednar, Susanne Granegger and Ulrike Horvath.

### References

ANDERSSON, B.S., BERAN, M., BAKIC, M. (1987). *In vitro* toxicity and DNA cleaving capacity of benzisoquinolinedione (nafidimide, NSC 308847) in human leukemia. *Cancer Res.*, **47**, 1040–1044.

CONSTANZE, M.E., KORZUN, A.H., HENDERSON, I.C., RICE, M.A., WOOD, W.C. & NORTON, L. (1989). Amonafide: an active agent in metastatic breast cancer (CALGB 8642). *Proc. Am. Soc. Clin. Oncol.*, **9**, 31 (A).

CRAIG, J. & CRAWFORD, E. (1989). Phase II trial of amonafide in advanced prostate cancer: a Southwest Oncology Group Study. *Proc. Am. Soc. Clin. Oncol.*, **8**, 147 (A).

ECKELMAN, W.A., REBA, R.A., GIBSON, R.E., REZESZOTARSKI, W.J., VIERAS, F., MAZAITIS, J.K. & FRANCIS, B. (1979). Receptor-binding radiotracers: a class of potential radiopharmaceuticals. *J. Nucl. Med.*, **20**, 350–357.

FARDE, L., HALL, H., EHRIN, E. & SEDVALL, G. (1986). Quantitative analysis of D2 dopamine receptor binding in the living brain by PET. *Science*, **231**, 258–261.

KRENNING, E.P., BAKKER, W.H. & BREEMAN, W.A.P. (1989). Localisation of endocrine-related tumours with radioiodinated analogue of somatostatin. *Lancet*, **i**, 242–245.

KUDO, M., VERA, D.R., TRUDEAU, W.L., STADALNIK, R.C. (1991). Validation of *in vivo* receptor measurements via *in vitro* radioassay: technetium-99m-galactosyl-neoglycoalbumin as prototype model. *J. Nucl. Med.*, **32**, 1177–1182.

LOGAN, J., WOLF, A.P., SHIUE, C.Y., FOWLER, J.S. (1987). Kinetic modeling of receptor-ligand applied to positron emission tomographic studies with neuroleptic tracers. *J. Neurochem.*, **48**, 73–83.

MARSHALL, J.S. & STOCKERT, W. (1978). Serum inhibitors of desialylated glycoproteins binding to hepatocyte membranes. *Biochim Biophys Acta*, **543**, 41–53.

PAVLIK, E.J., NELSON, K. & GALLION, H.H. (1990). Characterization of high specific activity (16α-$^{123}$I)iodo-17β-estradiol as an estrogen receptor-specific radioligand capable of imaging estrogen receptor-positive tumors. *Cancer Res.*, **50**, 7799–7805.

SCHATTEN, C., PATEISKY, I., VAVRA, N., EHRENBÖCK, P., ANGELBERGER, P., SIVOLAPENKO, G. & EPENETOS, A. (1990). Lymphscintigraphy with $^{123}$I-labeled epidermal growth factor. *Lancet*, **i**, 395–396.

SCHEITHAUER, W., DITTRICH, C., KORNEK, G., HAIDER, K., LINKESCH, W., GISSLINGER, C. & DEPISCH, D. (1991). Phase II study of amonafide in advanced breast cancer. *Breast Cancer Res.*, **20**, 63–67.

STADALNIK, R.C., VERA, D.R., WOODLE, E.S. (1985). Technetium-99m NGA functional hepatic imaging: preliminary clinical experience. *J. Nucl. Med.*, **26**, 1233–1242.

STEER, C.J. & ASHWELL, G. (1980). Studies on a mammalian hepatic binding protein specific for asialoglycoproteins: evidence for receptor recycling in isolated rat hepatocytes. *J. Biol. Chem.*, **255**, 3008–3013.

STOCKERT, R.J. & BECKER, F.F. (1980). Diminished hepatic binding protein for desialylated glycoproteins during chemical hepatocarcinogenesis. *Cancer Res.*, **40**, 3632–3637.

STOCKERT, J.J. & MORELL, A.G. (1983). Hepatic binding protein: the galactose receptor of mammalian hepatocytes. *Hepatology*, **3**, 750–757.

STRAUSS, G.L., CLORIUS, J., FRANK, T. & KAICK, G. (1984). Single photon emission computerized tomography (SPECT) for estimates of liver and spleen volume. *J. Nucl. Med.*, **25**, 81–85.

TAUXE, W.N., SOUSSALINE, F., TODD-POKROPEK, A., CAO, A., COLLARD, P., RICHARD, S., RAYNOUD, C. & ITTI, R. (1982). Determination of organ volume by single-photon emission tomography. *J. Nucl. Med.*, **23**, 984–987.

VERA, D.R., KROHN, K.A., STADALNIK, R.C. & SCHEIBE, P.O. (1984). Tc-99m-galactosyl-neoglycoalbumin: *in vivo* characterization of receptor-mediated binding to hepatocytes. *Radiology*, **151**, 191–196.

VERA, D.R., KROHN, K.A. & SCHEIBE, P.O. (1985). Identifiability analysis of an *in vivo* receptor binding radiopharmacokinetic system: IEEE. *Trans. Biomed. Eng.*, **32**, 312–322.

VERA, D.R., STADALNIK, D.R., TRUDEAU, W.L., SCHEIBE, P.O. & KROHN, K.A. (1991*a*). Measurement of receptor concentration and forward binding rate constant via radiopharmacokinetic modeling of $^{99m}$Tc-galactosyl-neoglycoalbumin. *J. Nucl. Med.*, **32**, 1169–1176.

VERA, D.R., WOODLE, E.S. & STADALNIK, R.C. (1991*b*). Kinetic senstivity of a receptor-binding radiopharmaceutical: technetium-99m galactosyl-neoglycoalbumin. *J. Nucl. Med.*, **30**, 1519–1530.

VIRGOLINI, I., ANGELBERGER, P., MÜLLER, C., BERGMANN, H. & SINZINGER, H. (1989*a*). $^{99m}$Tc-neoglycoalbumin binding to human hepatic binding protein. *Br. J. Clin. Pharmacol.*, **29**, 207–212.

VIRGOLINI, I., MÜLLER, C., KLEPETKO, W., BERGMANN, H., ANGELBERGER, P. & SINZINGER, H. (1989*b*). Decreased hepatic function in patients with liver metastases and hepatoma as monitored by $^{99m}$Tc-galactosyl-neoglycoalbumin. *Br. J. Cancer.*, **61**, 937–941.

VIRGOLINI, I., MÜLLER, C., ANGELBERGER, P., BERGMANN, H. & SINZINGER, H. (1991). Functional liver imaging with $^{99m}$Tc-galactosyl-neoglycoalbumin (NGA) in alcoholic liver cirrhosis and fibrosis. *Nucl. Med. Commun.*, **12**, 507–517.

VIRGOLINI, I., MÜLLER, C., HÖBART, J., SCHEITHAUER, W., ANGELBERGER, P., BERGMANN, H. & SINZINGER, H. (1992). Liver function in acute viral hepatitis as determined by a hepatocyte-specific ligand: $^{99m}$Tc-galactosyl-neoglycoalbumin. *Hepatology*, **15**, 593–598.

WAGNER, H.N., BURNS, H.D. & DANNALS, D.F. (1983). Imaging of dopamine receptors in the human brain by positron tomography. *Science*, **221**, 1264–1266.

WARING, M.J., ONZALEZ, A., JIMENEZ, A. (1979). Intercalative binding to DNA of antitumor drugs derived from 3-nitro-1,8-naphthalic acid. *Nucleic Acids Res.*, **7**, 217–223.

WOODLE, E.S., VERA, D.R., STADALNIK, R.C. & WARD, R.E. (1987). c-NGA imaging in liver transplantation: preclinical studies. *Surgery*, **102**, 55–62.